# Supplementary material for: RET receptor tyrosine kinase promotes breast cancer metastasis to the brain and RET inhibitors pralsetinib and selpercatinib suppress breast cancer brain metastases
Source: Cell Death Dis. 2026 Jun 1;17(1):679. doi: 10.1038/s41419-026-08890-z (PMC13433813; doi:10.1038/s41419-026-08890-z)
Supplement: Supplementary file 1 [file 41419_2026_8890_MOESM1_ESM.pdf]

Supplemental Figures

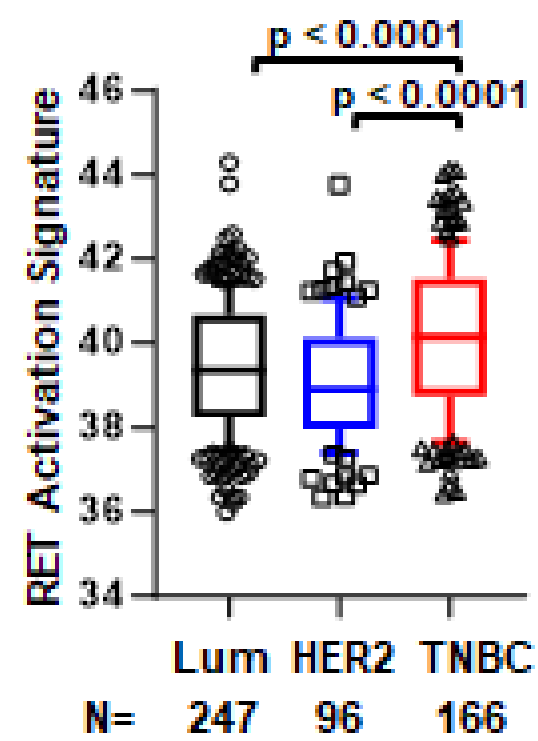

Supplemental Figure 1: RET activation signature is significantly higher in patients with TNBC subtype. To further investigate the clinical relevance of these observations, we conducted data mining analysis using our curated GEO breast cancer patient's dataset and analyzed RET activation across breast cancer subtypes.

		IC50 (μM)	
Subtype	Cell line	Pralsetinib	Selpercatinib
HER2-enriched	SKBR3	9.63	27.48
	SKBRM	9.97	25.80
TNBC	MDA-231	19.79	20.8
	MDA-231-BrM	12.09	29.53
	CN34	10.80	19.87
	CN34-BrM	18.04	28.07

Supplemental Figure 2: Breast cancer cells and their brain-tropic variants are sensitive to RET inhibitors (RETi). Representative IC50 values of RETi derived from treatment of HER2-enriched parental SKBR3 cell line and its brain-tropic variant SKBRM, and the TNBC cell lines MDA-MB-231 or CN34 and their brain-tropic variants MDA-MB-231-BrM or CN34-BrM, respectively.
