# Supplementary material for: RET receptor tyrosine kinase promotes breast cancer metastasis to the brain and RET inhibitors pralsetinib and selpercatinib suppress breast cancer brain metastases
Source: Cell Death Dis. 2026 Jun 1;17(1):679. doi: 10.1038/s41419-026-08890-z (PMC13433813; doi:10.1038/s41419-026-08890-z)
Supplement: Supplementary file 2 [file 41419_2026_8890_MOESM2_ESM.pdf]

Supplementary Table: RET Gene Activation Signature

ARTN
DOK1
DOK2
DOK4
DOK5
DOK6
FRS2
GAB1
GAB2
GDNF
GFRA1
GFRA2
GFRA3
GFRA4
GRB10
GRB2
GRB7
IRS2
MAPK7
NRTN
PDLIM7
PIK3CA
PIK3CB
PIK3CD
PIK3R1
PIK3R2
PIK3R3
PLCG1
PRKACA
PRKACB
PRKACG
PRKCA
PSPN
PTPN11
RAP1GAP
RET
SHC1
SHC3
SOS1
SRC
