# Supplementary material for: RET receptor tyrosine kinase promotes breast cancer metastasis to the brain and RET inhibitors pralsetinib and selpercatinib suppress breast cancer brain metastases
Source: Cell Death Dis. 2026 Jun 1;17(1):679. doi: 10.1038/s41419-026-08890-z (PMC13433813; doi:10.1038/s41419-026-08890-z)

Original WB Images

Fig. 2A

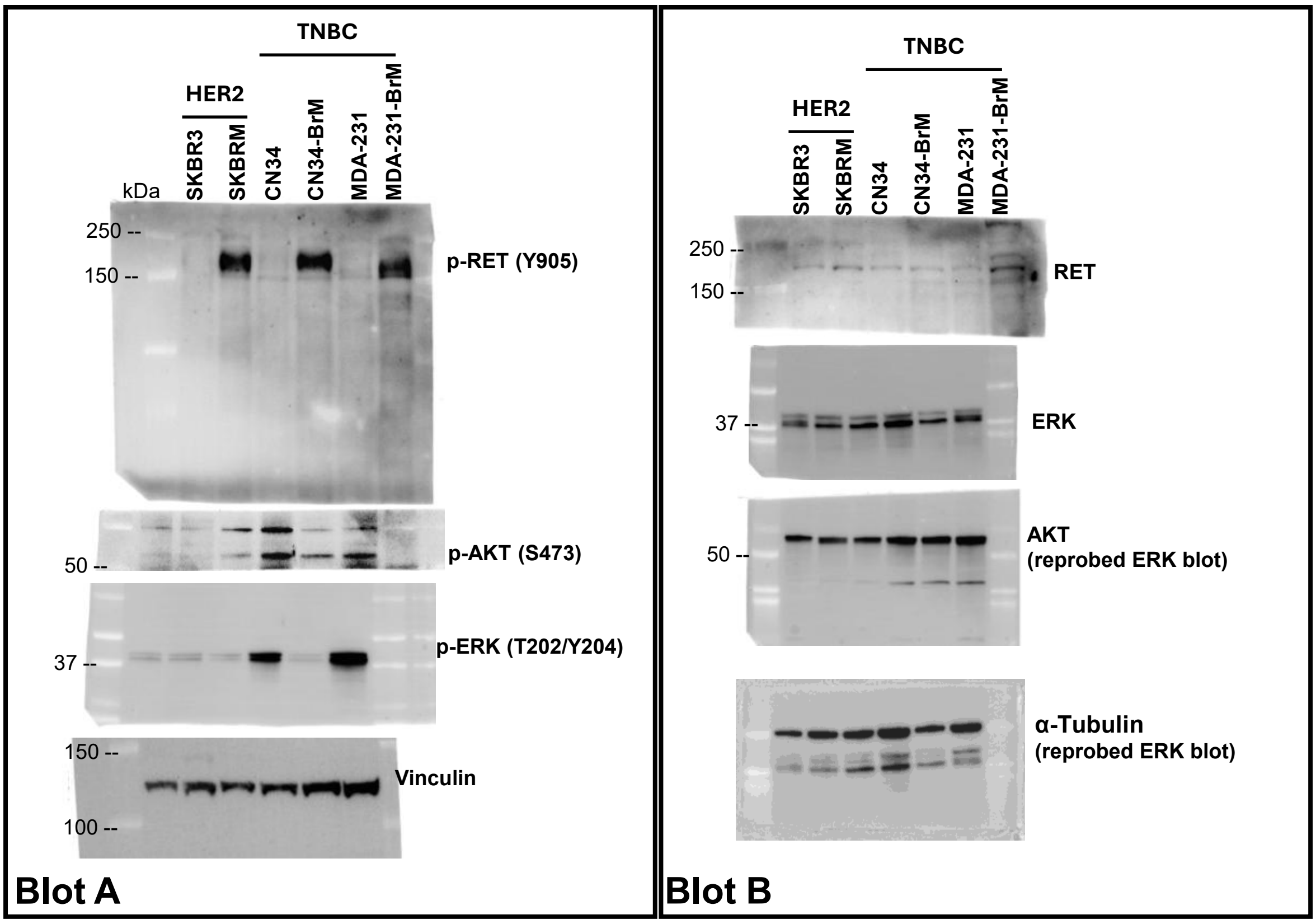

Original WB Images

Fig. 3B

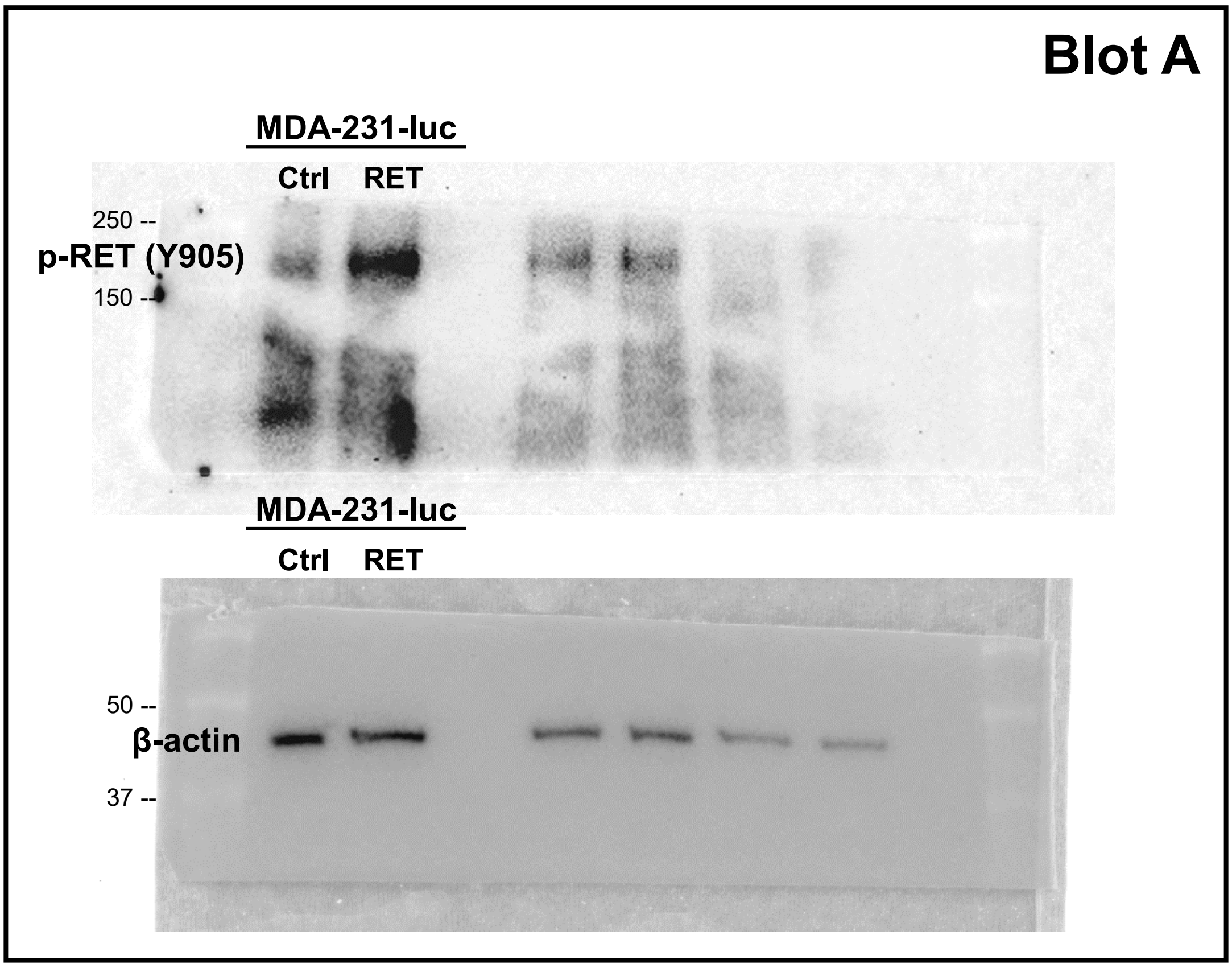

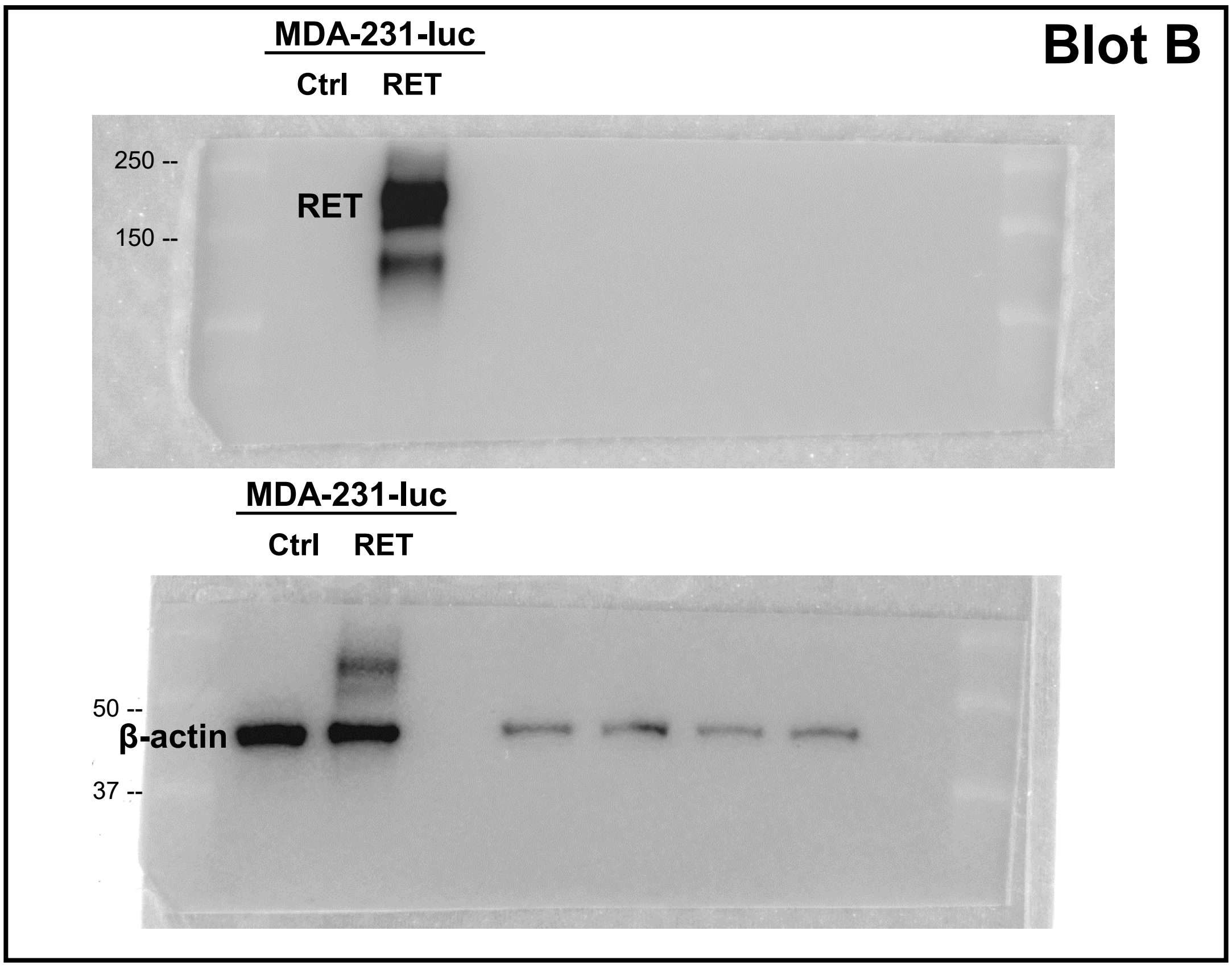

Original WB Images

Fig. 5C

Pralsetinib-treated samples

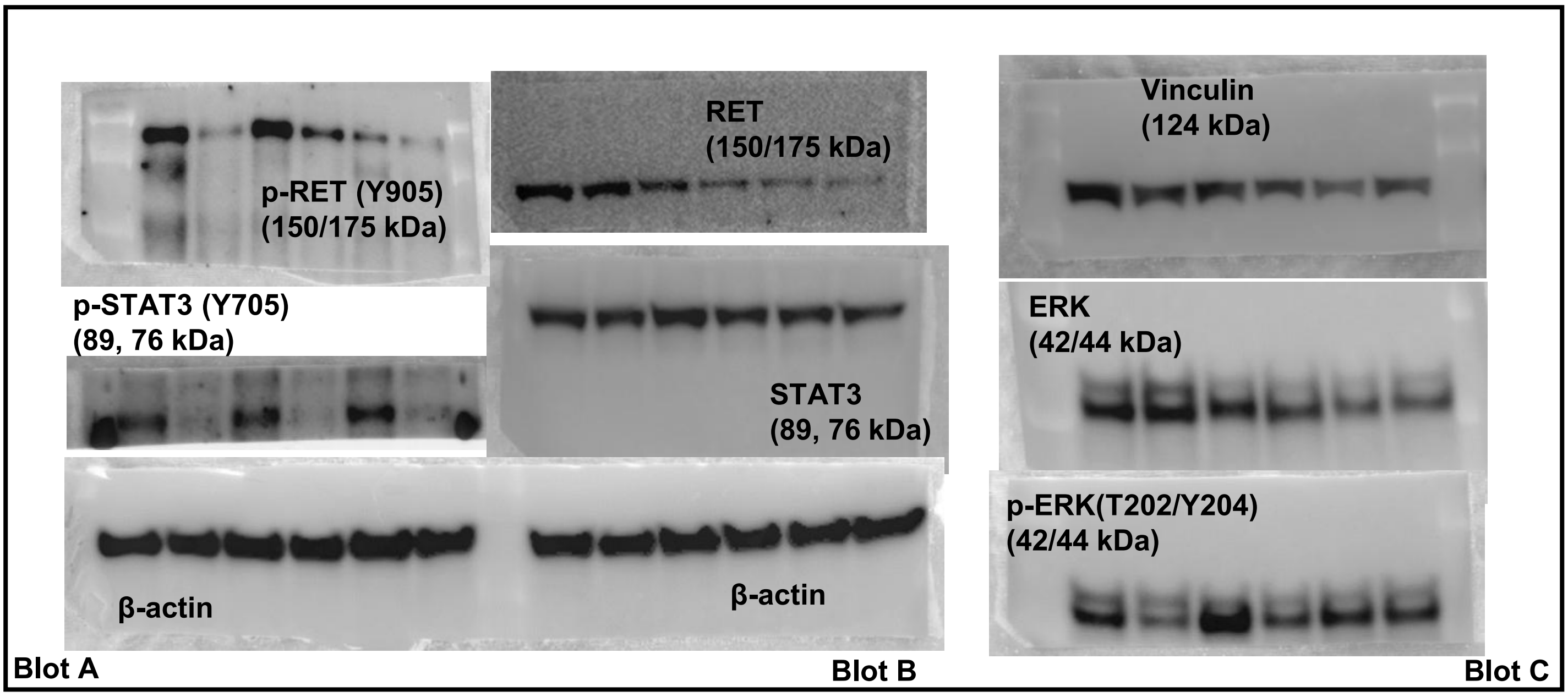

Selpercatinib-treated samples

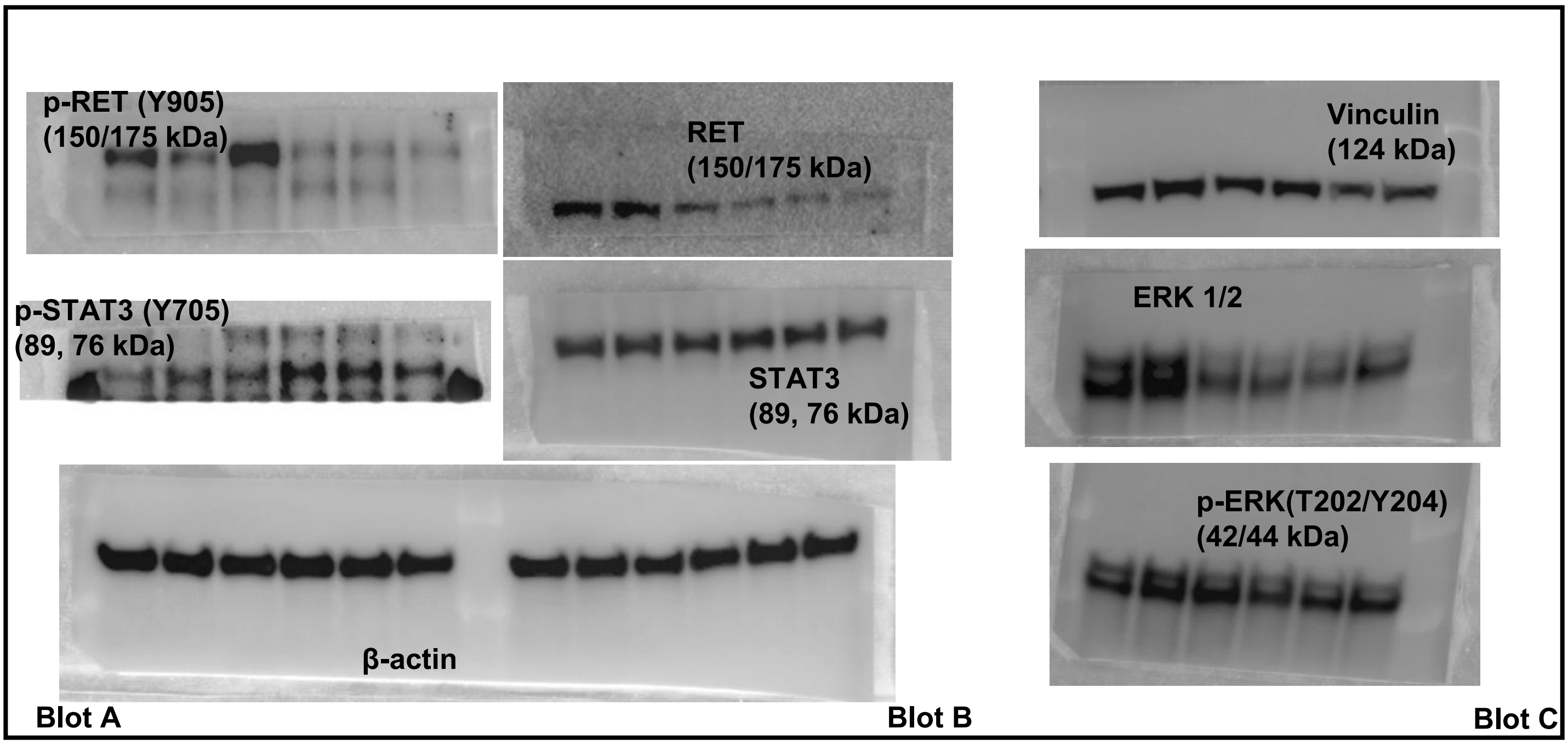

Original WB Images

Fig. 5G/5H

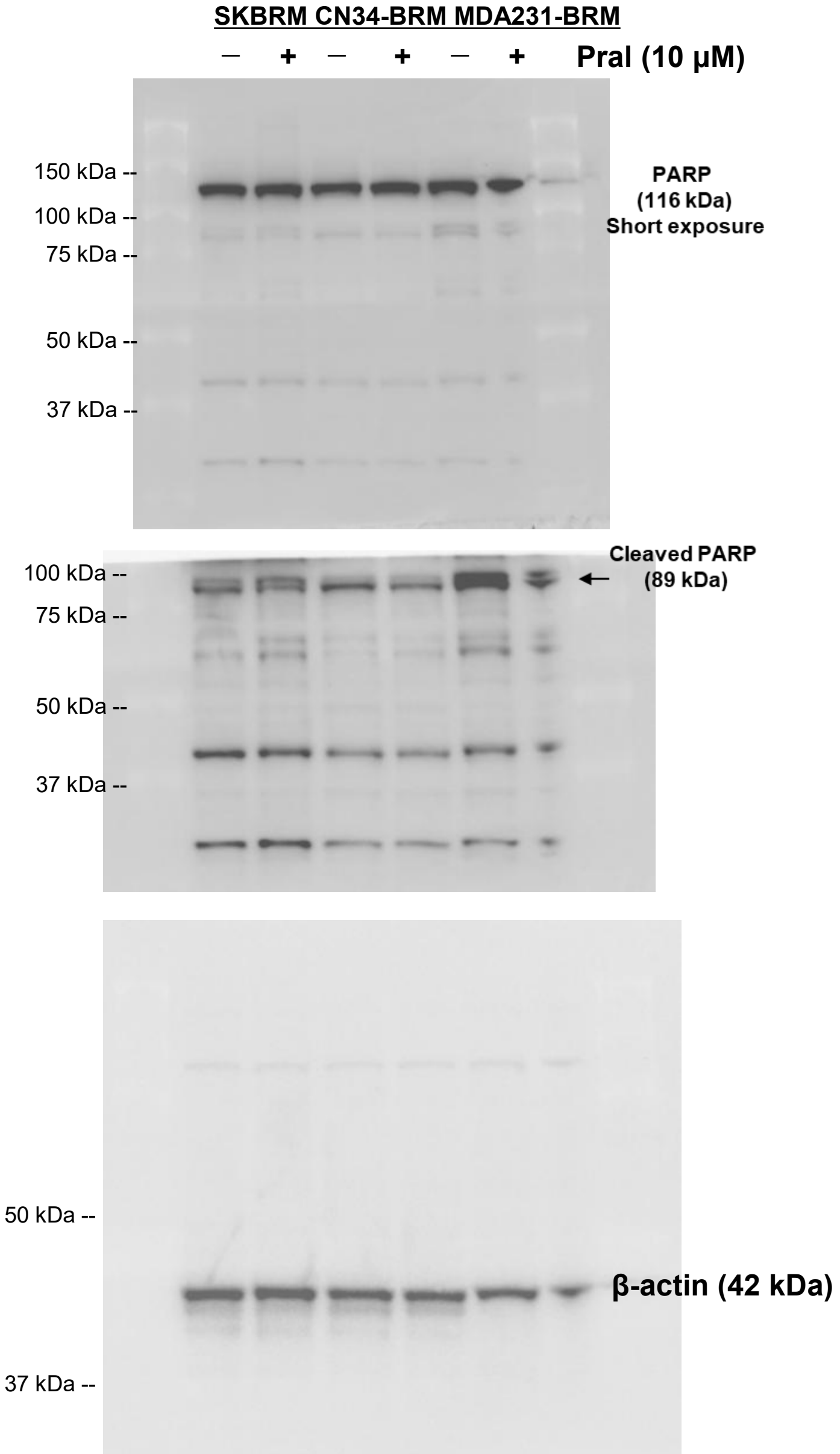

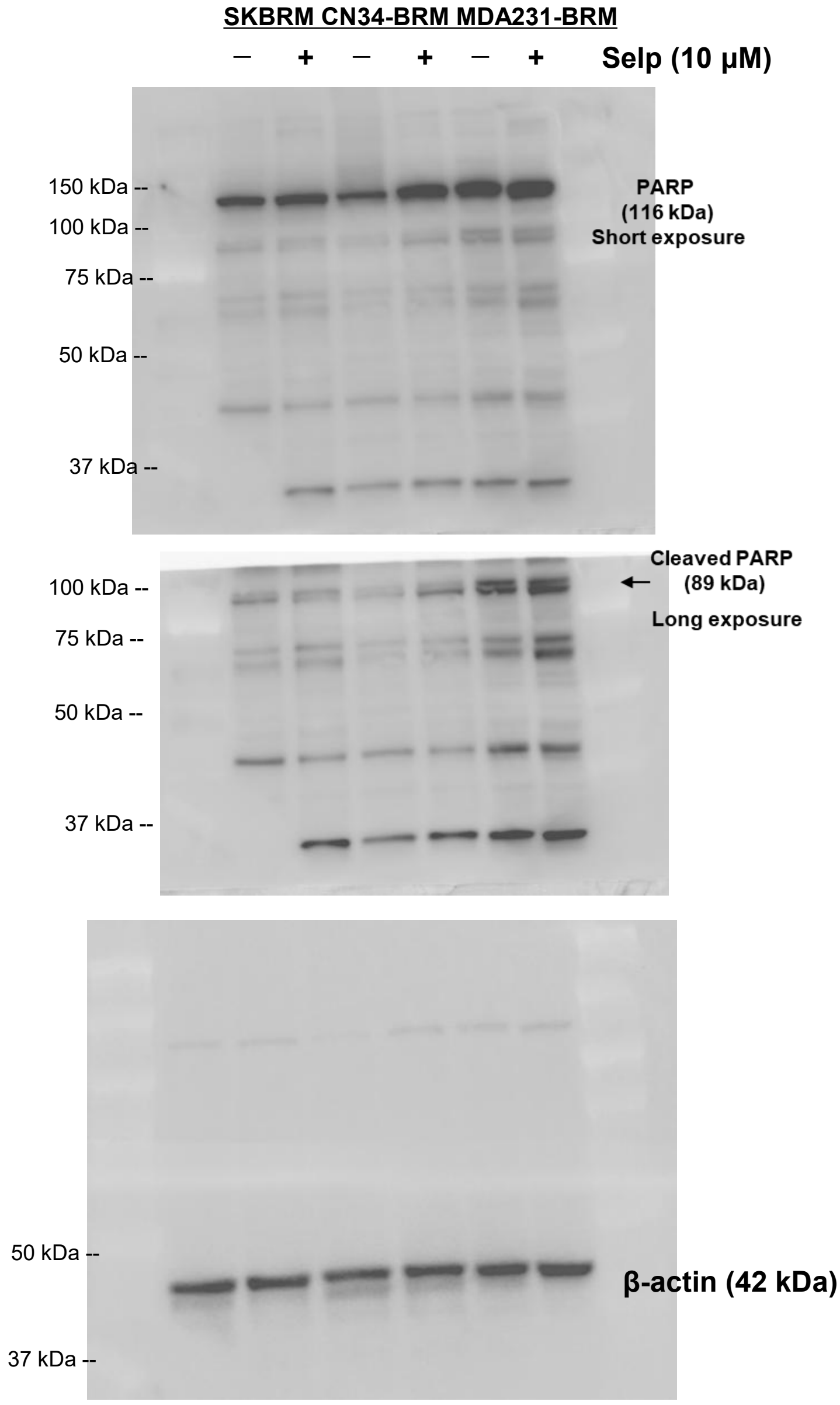

Supplement: Supplementary file 3 [file 41419_2026_8890_MOESM3_ESM.pdf]
